# Thioflavin T fluoresces as excimer in highly concentrated aqueous solutions and as monomer being incorporated in amyloid fibrils

**DOI:** 10.1038/s41598-017-02237-7

**Published:** 2017-05-19

**Authors:** Anna I. Sulatskaya, Andrey V. Lavysh, Alexander A. Maskevich, Irina M. Kuznetsova, Konstantin K. Turoverov

**Affiliations:** 1 Laboratory of Structural Dynamics Stability and Folding of Proteins, Institute of Cytology of the Russian Academy of Sciences, St. Petersburg, 194064 Russia; 2 Department of Physics, Yanka Kupala Grodno State University, Grodno, 230023 Belarus; 3 Department of Biophysics, Peter the Great Saint-Petersburg Polytechnic University, St. Petersburg, 195251 Russia

## Abstract

Fluorescence of thioflavin T (ThT) is a proven tool for amyloid fibrils study. The correct model of ThT binding to fibrils is crucial to clarify amyloid fibrils structure and mechanism of their formation. Although there are convincing evidences that ThT has molecular rotor nature, implying it’s binding to fibrils in monomer form, speculations concerning ThT binding to fibrils in aggregated forms appear in literature so far. The elaborated approach for fluorescence intensity correction on the inner filter effects applied to ThT aqueous solutions with a wide range of concentration allowed characterizing ThT excimers fluorescence and showing its difference from that of ThT bound to fibrils. Obtained results experimentally prove the monomer model of ThT binding to amyloid fibrils and demonstrate wide capacity of the used approach in the spectroscopy of other fluorescent dyes for examination of concentration self-quenching and deformation of fluorescence spectra, dye molecules interaction, dimers and excimers formation.

## Introduction

Thioflavin T (ThT) fluorescence is a proven method to detect the amyloid fibrils that frequently accompany the development of serious human diseases, including Alzheimer’s, Parkinson’s, prion, and many other diseases^1, 2^. Over the past 5–10 years, the application of ThT has been extended beyond simply testing for the presence of amyloid fibrils; instead, ThT has been used to study the structure of amyloid fibrils and the mechanism by which they form. These studies are important not only for molecular medicine but also for elucidating protein folding and misfolding, as well as for the materials science applications related to the exceptional strength of amyloid fibrils^3, 4^.

Obviously, the success of ThT use for studying amyloid fibrils largely depends on how well its photophysical properties and its mechanism of interaction with amyloid fibrils are understood. Most researchers hold the view that ThT incorporates into amyloid fibrils in the monomeric form. Furthermore, it is thought that the main factor contributing to the significant (in some cases, several thousand-fold) increase in its fluorescence quantum yield upon binding to fibrils is a restriction of the internal rotations of the ThT benzothiazole and aminobenzene rings relative to one another in the excited state^5^. These ideas were first proposed in the work by Voropay *et al*.^6^. Thereafter, this assertion was proven and developed using quantum chemical calculations of the ThT molecule in the ground and excited states^7^, examination of the dependence of the ThT fluorescence quantum yield on the solution viscosity, pressure and temperature^8–11^, and studies of the picosecond decay kinetics of ThT fluorescence in solutions with different dielectric properties and viscosities^5, 9–14^. Based on the molecular rotor nature of ThT, a model of its incorporation into amyloid fibrils was proposed^15^.

However, there is another view on the ThT fluorescence intensity increase upon amyloid fibrils binding. In this model, ThT incorporates into amyloid fibrils in an aggregated form, and these ThT aggregates are thought to have a high fluorescence quantum yield. This suggestion grew from early work^16^ where a weak, short wavelength fluorescence band (λ_ex_ = 340–350 nm, λ_em_ = 440 nm) was erroneously attributed to free ThT in solution, and an intense, long wavelength fluorescence band was assigned to ThT aggregates bound to the amyloid fibrils (λ_ex_ = 412 nm, λ_em_ = 490 nm). As we have previously shown that work and subsequent papers^17, 18^ ignored the fact that the short wavelength band of the ThT fluorescence excitation spectrum does not coincide with the absorption spectrum of the dye, as it must be^19^. On the contrary, the maximum of the fluorescence excitation spectrum of this spectral band corresponds to a minimum in the absorption spectrum. Consequently, the short wavelength band of the ThT fluorescence spectrum in aqueous solutions requires a special explanation, not the long wavelength band. Later, the short wavelength spectral band of ThT in alcohol and in aqueous solutions was explained based on the results of quantum chemical calculations using the molecular rotor model to describe the nature of ThT^19–24^. Despite the invalidity of the assumptions in which the intense fluorescence of ThT bound to amyloid fibrils was assigned to aggregates, some authors still refer to these early works without introducing new evidence to justify the adoption of this model^22, 25^.

The groundlessness of the idea that ThT binds to amyloid fibrils in micelle form^18^ was shown spectroscopically^26^ and by atomic force microscopy^27^. In some works it was shown that in conditions which provoke ThT molecules interaction with each other they form small aggregates^28–31^. It was found that the fluorescence spectrum of the ThT excimers is significantly red shifted as compared to that of ThT monomers and radically differs from that of ThT bound to amyloid fibrils. After that it seems that issue whether ThT bound to amyloid fibrils is determined by ThT excimer is closed. Nevertheless, the papers with assumption that fluorescence of ThT bound to amyloid fibrils is determined by its excimers still appear^22, 25, 32^. The present work aims to experimentally show the possibility of ThT excimer formation in high concentration aqueous solution and show that this fluorescence differs dramatically from fluorescence of ThT bound to amyloid fibrils^19^.

## Results and Discussion

### Concentration dependence of ThT fluorescence

**Figure 1:**
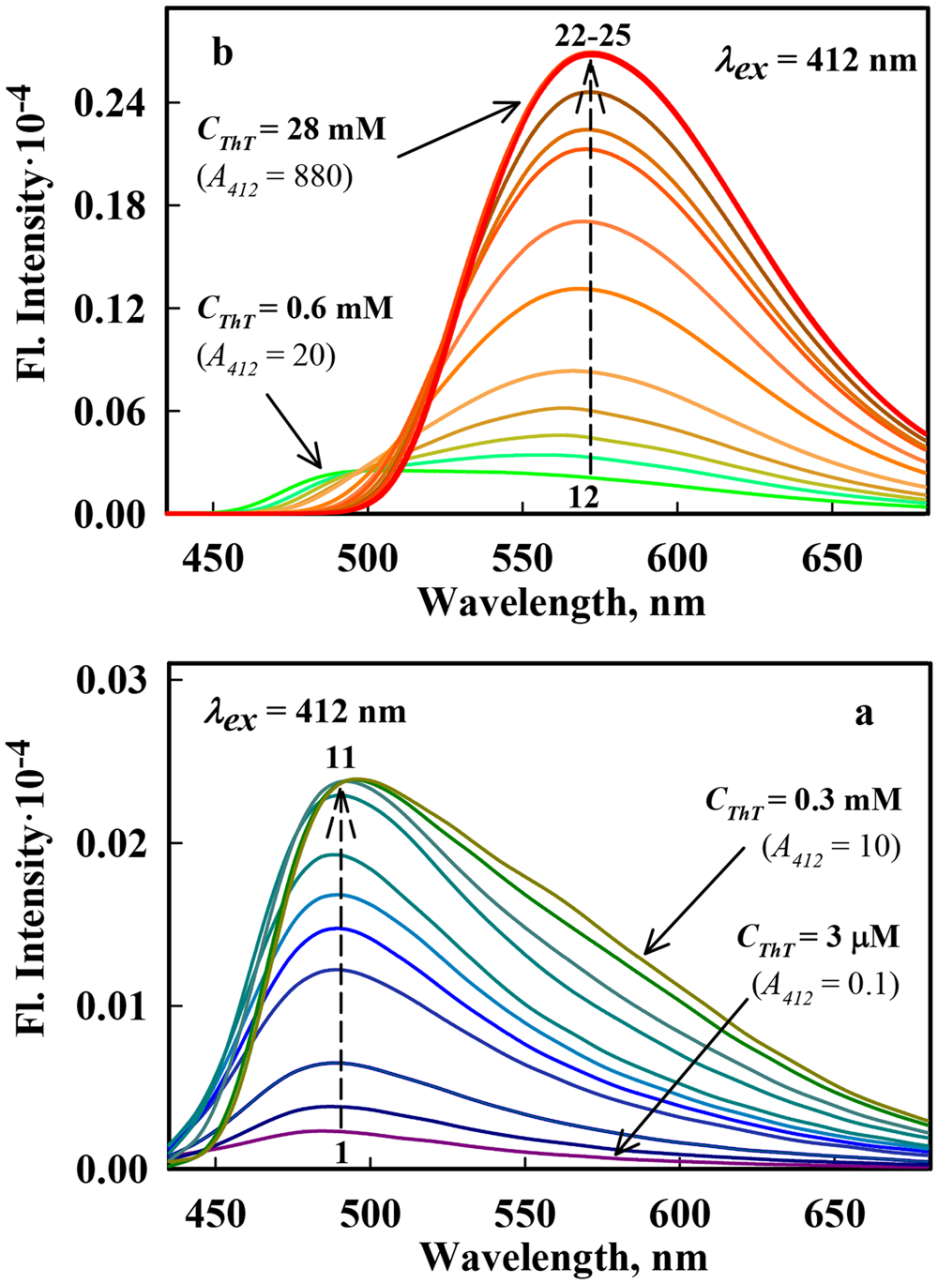
Fluorescence spectra of ThT in aqueous solutions with different concentrations. Fluorescence spectra before corrections for the effect of the primary and secondary inner filter effects, as recorded using a Cary Eclipse spectrofluorimeter (Right Panels). Panel a: The ThT concentrations increases from 3 μM to 0.3 mM (curves (1)–(11) correspond to ThT concentrations 3, 6, 10, 16, 22, 29, 60, 110, 190, 220, 320 µM, respectively), Panel b: The ThT concentration increases from 0.6 mM to 28 mM (curves (12)–(25) correspond to ThT concentrations 0.6, 0.8, 1.1, 1.4, 1.7, 1.9, 2.2, 2.5, 2.8, 6, 13, 19, 25, 28 mM, respectively). The excitation wavelength was 412 nm.

**Figure 2:**
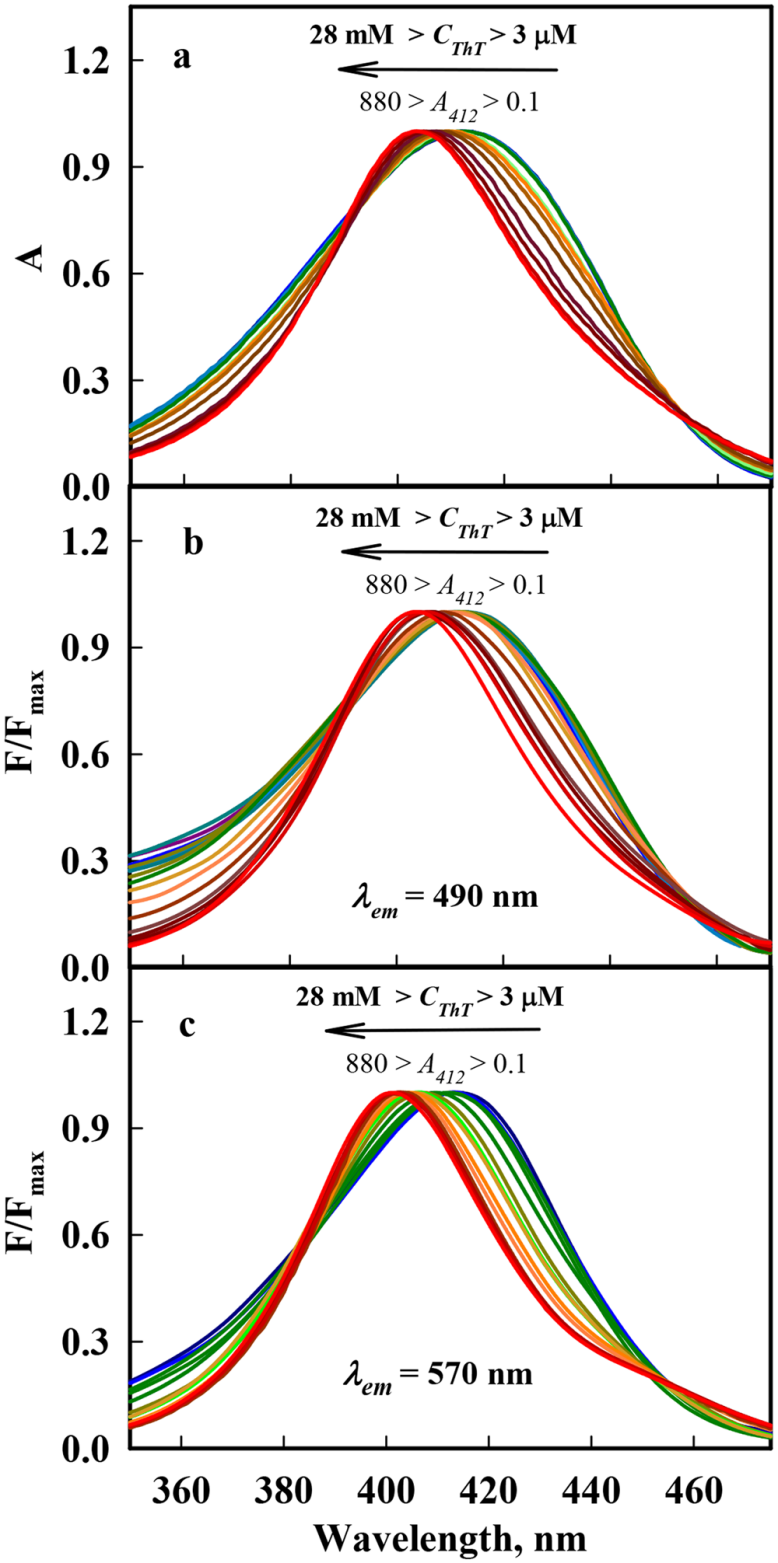
Absorbance spectra (Panel a) and fluorescence excitation spectra recorded at *λ*_*em*_ = 490 nm (Panel b) and at *λ*_*em*_ = 570 nm (Panel c) of ThT aqueous solutions with concentrations ranging from 3 μM to 28 mM, which correspond to absorbance of 0.1 to 880 at 412 nm. We used the same colors of the absorption and fluorescence excitation spectra of the solutions with concentration of ThT equal to concentration of the solutions fluorescence spectra of that are presented in Fig. 1.

To experimentally prove ThT excimer formation in aqueous solution, it was necessary to measure and compare spectral characteristics of the dye over a wide range of concentrations. The use of the Cary Eclipse spectrofluorimeter allowed us to reliably measure the fluorescence spectra of ThT in aqueous solution in a wide range of the dye concentrations: from 3·10^−6^ to 3·10^−2^ M (in the range of solution absorbance from 0.1 to 880) in the same conditions (Fig. 1). With increasing dye concentration, the short wavelength region of the fluorescence spectrum gradually shifts to emergence longer wavelengths and a new band appears in the long wavelength region of the spectrum. The contribution to the fluorescence spectrum of the long wavelength component becomes dominant when the dye concentration is greater than 8 · 10^−4^ M (*A* = 25). Thus the increase in ThT concentration from 3·10^−6^ to 3·10^−2^ M leads to the substantial red shift of its fluorescence spectra (maximum is shifted from 490 to 570 nm). These changes in ThT concentration also lead a slightly blue shift of its absorption spectra (Fig. 2), that can be explained by ThT excimers formation.

**Figure 3:**
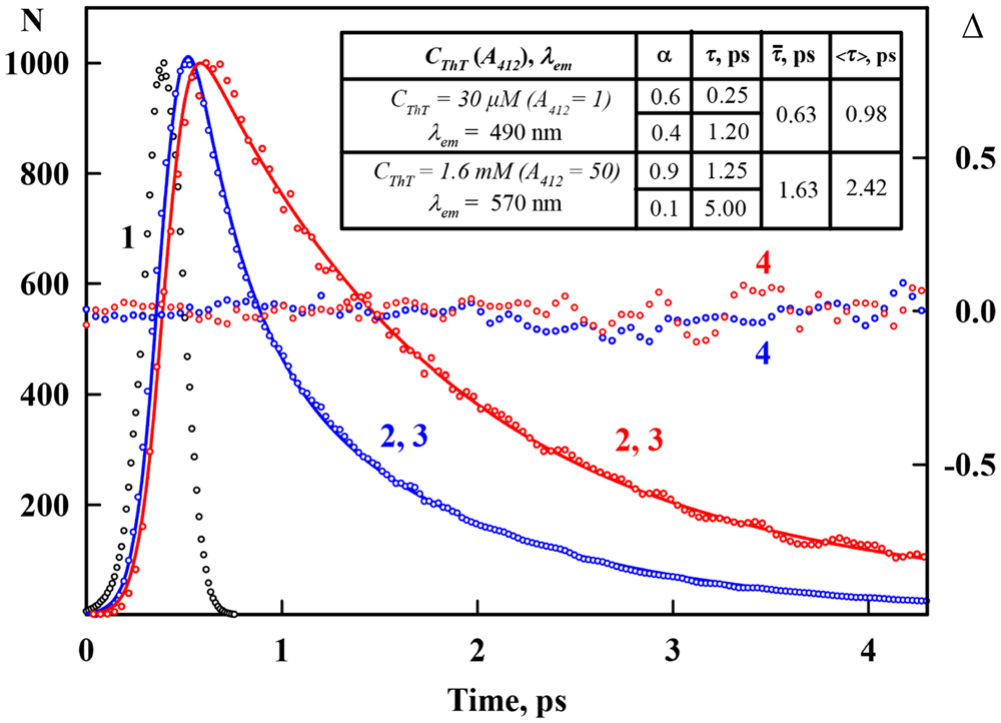
Decay curves of ThT fluorescence in aqueous solution. The excitation lamp profile (black dotted curve (1)), experimental decay curves of the fluorescence of ThT solutions with a concentration of 32 μM (*A*_*412*_ = 1) recorded at 490 nm (blue dotted curve (2)) and with a concentration of 1.6 mM (*A*_*412*_ = 50) recorded at 570 nm (red dotted curve, 2), best-fit calculated fluorescence decay curves (blue (3) and red (3) straight curves, respectively), and the deviation between the experimental and calculated decay curves (blue (4) and red (4) dotted curves, respectively) are shown. The samples were excited with second harmonic light (420 nm), and the 490 and 570 nm fluorescence from the samples was up-converted by mixing it with the fundamental light pulse (gate pulse ~840 nm). The fluorescence decay curves show the best fit to a biexponential decay model. The calculated values of average lifetime $\overline{\tau }=\sum {\alpha }_{i}{\tau }_{i}$ and root mean square lifetime $\langle \tau \rangle =\frac{\sum {\alpha }_{i}{\tau }_{i}^{2}}{\sum {\alpha }_{i}{\tau }_{i}}$ are presented.

In the work by Froehlich and Wehry^33^ two different possibilities of excimer and exciplexes appearance is discussed: 1) complex is formed by the interaction of an excited molecule (M*) with unexcited one (M in the case of excimer or different molecule Q in the case of exciplex): M* + M → (M_2_)* and M* + Q → (MQ)*; 2) complex between monomers is formed in the ground state: MM + hυ → (M_2_)* and MQ + hυ → (MQ)*. As the increase in ThT concentration leads not only to the significant red shift of fluorescence spectrum but also to the small but reliably recorded blue shift of absorption spectrum, one can conclude that the ThT molecules interact before excitation, i.e. the second scheme of excimers formation is realized. Furthermore, the first scheme of excimers formation cannot be realized because the lifetime of the excited state is very small, approximately 1 ps (Fig. 3). Important, fluorescence excitation spectra recorded at 490 nm and at 570 nm are close to the corresponding absorption spectrum (Fig. 2), proving that the red fluorescence (excimers fluorescence) is determined by the same ensemble of molecules which determines ThT absorption band.

Figure 1 also shows that with the increase in ThT concentrations the short wave front of fluorescence spectra became steeper. The reason of it became clear from the Fig. S2 (Left panels), where except fluorescence spectra the long wavelength region of the absorption spectra (dotted lines) are shown. So it became evident that the change in the fluorescence spectrum shape in the short wavelength region is caused by the absorption of the fluorescence; this change is referred to as the secondary inner filter effect. In principle, the reabsorption of fluorescence light quanta can lead to secondary fluorescence, but due to the very low fluorescence quantum yield of ThT in aqueous solution (*q* = 10^−4^)^21^, the contribution of secondary fluorescence to the emission, which is proportional to *q* ^*2*^, is negligibly small.

### Monomer and excimer fluorescence spectra undistorted by the secondary inner filter effect

**Figure 4:**
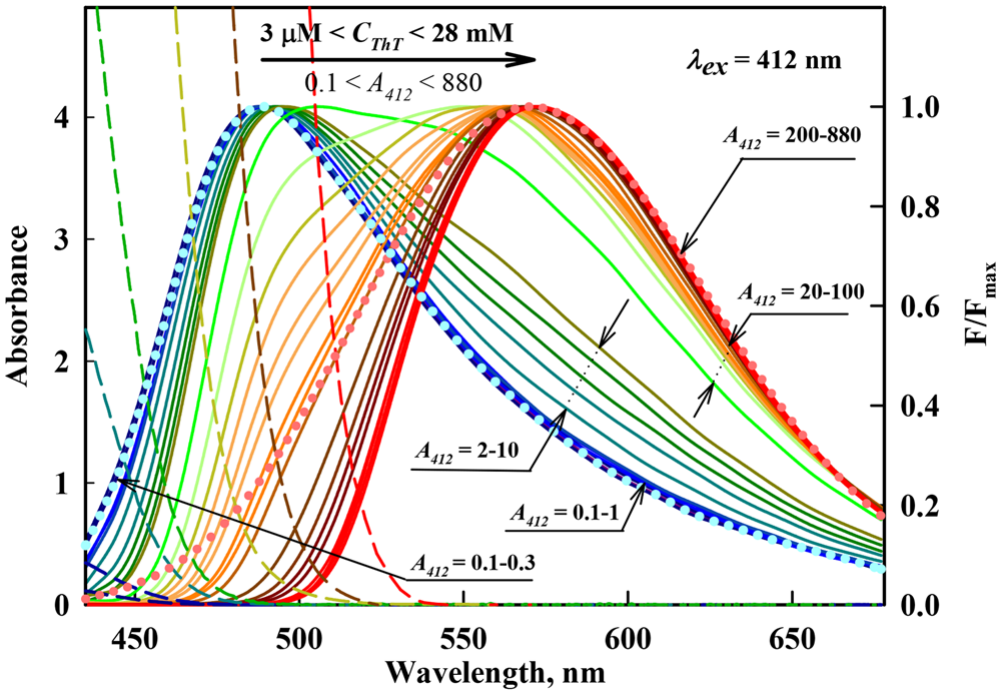
The fluorescence spectra of aqueous solutions of ThT at different concentrations with corrections for the primary inner filter effect and normalization at the spectral maxima. The ThT concentration was increased from 3 μM to 28 mM, which corresponds to an absorbance change from 0.1 to 880. *λ*_*ex*_ = 412 nm. The forms of the monomer and excimer fluorescence spectra are given in blue and pink dots, respectively. The dashed curves represent the long wavelength edge of the absorption spectra of ThT solutions with concentrations 0.006, 0.016, 0.11, 0.32, 1.1, 6, 28 mM. Curves with *А*_412_ = 0.1–0.3 correspond to ThT concentrations 3, 6, 10 µM, curves with *А*_412_ = 0.1–1 correspond to ThT concentrations 3, 6, 10, 16, 22, 29 µM, curves with *А*_412_ = 2–10 correspond to ThT concentrations 0.06, 0.11, 0.19, 0.22, 0.32 mM, curves with *А*_412_ = 20–100 correspond to ThT concentrations 0.6, 0.8, 1.1, 1.4, 1.7, 1.9, 2.2, 2.5, 2.8 mM, curves with *А*_412_ = 220–880 correspond to ThT concentrations 6, 13, 19, 25, 1.7, 28 mM.

Figure 4 shows fluorescence spectra reduced to unity at the maximum, which represent the superposition of the fluorescence spectra of both dye monomers and excimers that are distorted in the short wavelength range by the reabsorption of fluorescence (secondary inner filter effect).

The fluorescence spectra measured for solutions with concentrations of 3, 6 and 9 μM (absorbance *A*_*412*_ = 0.1, 0.2 and 0.3, respectively) do not differ in shape (Fig. 4). Thus, for these solutions, fluorescence reabsorption does not change the fluorescence emission spectrum in the short wavelength region. Furthermore, the excimer fluorescence must be negligible for such low absorbance. Thus, the fluorescence spectrum of solutions in this range of concentrations (absorbance) is determined solely by dye monomers, and consequently, the shape of these spectra can be used as a shape of the monomer ThT spectrum.

However, the shape of the fluorescence spectra of ThT solutions with the maximum dye concentration (3·10^−2^ M) cannot be considered to be the shape of an excimer spectrum because these spectra are significantly disturbed by reabsorption at the short wavelength edge (Fig. 4). At lower ThT concentrations, the influence of reabsorption decreases, but even at 3·10^−3^ M ThT (*A*_412_ = 100), the contribution of monomer fluorescence to the total fluorescence spectrum becomes detectible. Therefore, the excimer spectrum was approximated by a lognormal distribution function, which at the long wavelength edge, coincides with the fluorescence spectrum of ThT at high concentration (Fig. 4).

### Correction of fluorescence spectra on the secondary inner filter effect

On the basis of the known shapes of monomer and excimer spectra, each recorded fluorescence spectrum was decomposed into these two components (see Methods). The contribution of the monomer (*F*_*mon*_) and excimer (*F*_*exc*_) fluorescence in the recorded fluorescence spectra was determined in the spectral region where a fluorescence spectrum is not distorted to the secondary inner filter effect (λ_em_ > 550 nm). On the basis of the true shapes of monomer and excimer fluorescence spectra and their contribution to total fluorescence true fluorescence spectra (corrected for the secondary inner filter effect) where constructed.

**Figure 5:**
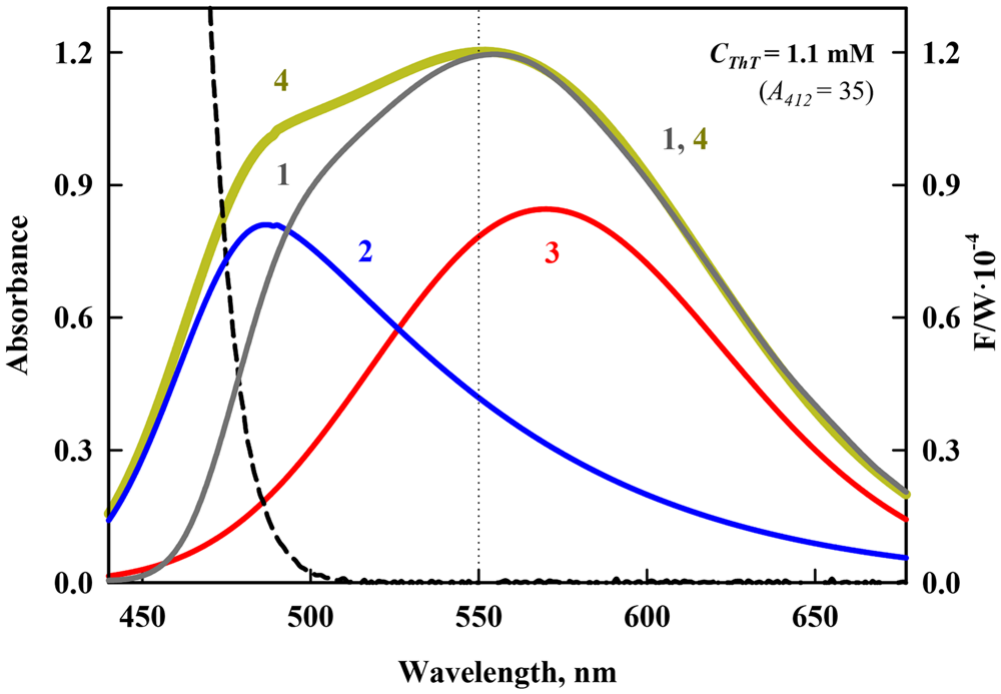
Decomposition of the fluorescence spectra into components and correction for the secondary inner filter effect. The experimentally recorded fluorescence spectrum corrected for the primary inner filter effect (curve 1), monomer and excimer fluorescence spectra (curves 2 and 3, respectively), and fluorescence spectrum of two components corrected for the primary and secondary inner filter effect (curve 4). The dashed curve represents the long wavelength edge of the absorption spectrum of a solution with a concentration of 1.1 mM.

Figure 5 shows the corrected for the primary inner filter effect fluorescence spectrum of solution with ThT concentration 1.1·10^−3^ M (curve 1), the contribution of the monomer and excimer fluorescence (curves 2 and 3) and total corrected spectrum (curve 4). The overall fluorescence spectra corrected for the primary and secondary inner filter effects of solutions with concentrations ranging from 3·10^−6^ to 3·10^−2^ M (absorbance *A*_*412*_ from 0.1 to 880) are shown in Supplementary Fig. S2, panels on the right.

### The monomer and excimer fractions of the total fluorescence intensity of ThT solutions of different concentrations

The gradual transformation of the spectra with increasing concentration of the dye and the possibility of dividing of spectra into two components with an obvious isobestic point (Supplementary Fig. S3) are arguments in favor of the presence of only two components that supports our conclusion of the formation of excimer of ThT molecules at high concentrations of the dye in aqueous solution.

**Figure 6:**
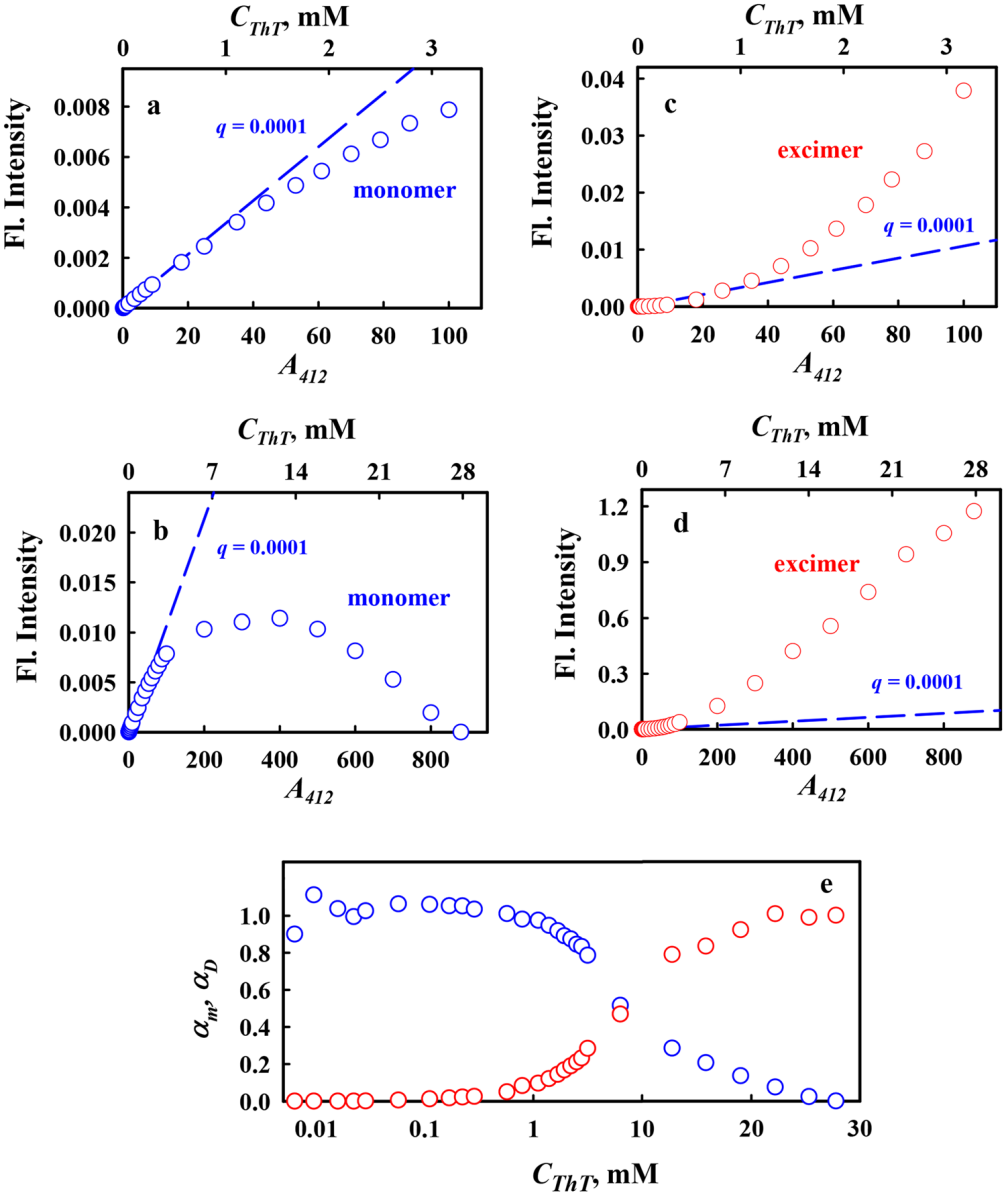
The dependences of monomers and excimers fractions of total fluorescence intensity of ThT solutions on the dye concentration. Panels a and b represent fluorescence of monomers fraction, Panels c and d represents fluorescence of excimers fraction. The fluorescence intensity was corrected for the inner filter effect. The dashed line represents the dependence calculated with the use of previously defined value for the ThT fluorescence quantum yield in an aqueous solution, 0.0001^8^. Panel e shows the dependence of monomers and excimers fractions in ThT solution on the dye concentration. The nonuniform abscissa scale was used: a logarithmic scale for *C*_*ThT*_ less than 10 mM, and a linear scale for *C*_*ThT*_ more than 10 mM. The half-maximum of the dependences corresponds to 7.3 mM ThT.

The dependence of the total fluorescence intensity with corrections for the primary and secondary inner filter effects on the dye concentration of monomers and excimers is given in Fig. 6. In the range of dye concentrations from 3·10^−6^ to 3·10^−4^ M (corresponding to absorbance values ranging from 0.1 to approximately 10) there is an almost linear dependence of the total fluorescence intensity on the concentration with a slope that is consistent with the previously defined value for the ThT fluorescence quantum yield in an aqueous solution, 0.0001^8^. As the dye concentration increases, the fluorescence intensity increases less rapidly; thus, the concentration dependence of the total fluorescence intensity of monomers is nonlinear (Fig. 6, Panels a and b). This effect is expected because in this range of the dye concentrations, the contribution of excimers fluorescence to the total emission increases (Fig. 6, Panels c and d).

The dependence of the fractions of ThT monomers and ThT excimers in solution (*α*_*mon*_ and *α*_*exc*_), upon the total concentration of ThT in solution (*C*_*0*_ = *C*_*mon*_ + 2*C*_*exc*_) was determined on the basis of fluorescence spectra corrected on the primary and secondary inner filter effects and their decomposition on the components, namely fluorescence spectra of monomers and excimers:

1 $${F}_{corr}={F}_{corr,mon}+{F}_{corr,exc}={A}_{mon}{q}_{mon}+{A}_{exc}{q}_{exc}$$

and

2 $${F}_{corr}/{C}_{0}={\alpha }_{mon}{\varepsilon }_{mon}{q}_{mon}+{\alpha }_{exc}{\varepsilon }_{mon}{q}_{exc}$$

Here *F*_*corr*_ is total fluorescence intensity corrected on the primary and secondary inner filter effects^34^; the subscripts *mon* and *exc* indicate on monomers and excimers, *A* and *q* are absorbance and fluorescence quantum yield. From the equality *ε*_*mon*_ and *ε*_*exc*_ it follows that ${q}_{exc}\approx 10{q}_{mon}$ (Fig. 6, Panel d).

Determined in this way dependences of α_mon_ and α_exc_ on *C*_*0*_ are presented in the Fig. 6, Panel e. To present the dependence in the range of small and large values of *C*_*0*_ the dependence is represented using nonuniform abscissa scale: a logarithmic scale when *C*_*0*_ is less than 10 mM, and a linear scale when *C*_*0*_ is more than 10 mM. The concentration of the dye at which *α*_*mon*_ = *α*_*exc*_ can characterize the ability of ThT to form excimers. This concentration equals to 7.3 mM. When ThT is used as a marker for the formation of amyloid fibrils its concentrations is sufficiently low, not exceeding 10 μM. At such concentration of ThT the contribution of excimers in total fluorescence can be neglected.

### ThT excimer photophysics

In the case of random molecule distribution, to estimate the concentration at which molecules may be in contact with one another, we determine the probability of two dye molecules (*N* = 2) being simultaneously located in a sphere with a radius equal to the long axis of the ThT molecule (16.2 Å) at different ThT concentrations using the Poisson equation ${\omega }_{N}={\overline{N}}^{N}\exp (-\overline{N})/N!$ ^35^. The results of this calculation indicate that assuming a random distribution of molecules in solution, the probability that ThT molecules directly contact each other is not very high even at the highest concentration of ThT. However, if interactions between ThT molecules are free energetically more favorable than interactions with solvent molecules, then the ThT molecules are not uniformly distributed and instead are clustered. The small but reliable blue shift of the absorption spectrum and significant red shift of fluorescence spectrum, which indicates the formation of excimers, prove this.

**Figure 7:**
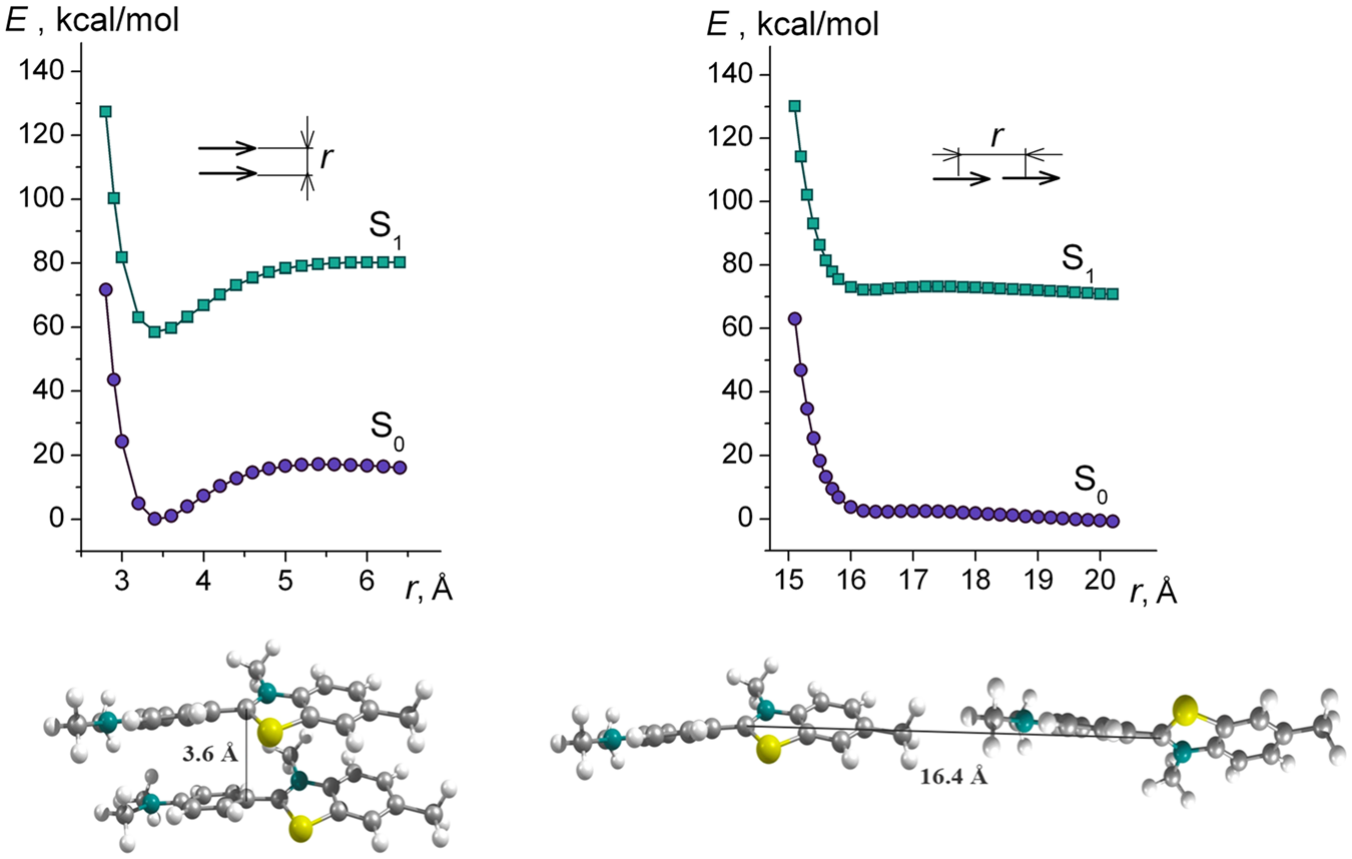
Quantum chemical calculations of the formation of ThT aggregates. It is shown that ThT molecule may form excimers in the form of a “sandwich” (left panel) and a “junction” (right panel) (H- and J-aggregates, respectively).

Quantum chemical calculations using density functional theory (DFT) show that the ThT molecule may form excimers in the form of a “sandwich” and a “junction” (H- and J-aggregates, respectively, Fig. 7). Earlier it was suggested that ThT molecules form J-aggregates with the angle between the dipole moments equal to 120°^32^. However, this type of molecular coupling is unstable, as demonstrated by the dependence of the total energy of the system on the distance between them: the curve has no minimum (Fig. 7).

On the contrary, for the pair of parallel to each other molecules forming the “sandwich” structure, such dependence has a pronounced energy minimum corresponding to a distance between the molecules *d* = 3.6 Å (Fig. 7). Calculations for ThT excimer with “sandwich” structure in which the molecules are antiparallel to each other showed that the dependence of *E(r)* is similar to that for excimer with a parallel arrangement of the molecules, however the energy value at the minimum less on 0.2 kcal/mole. This can be explained by the fact that for different configurations of excimer the energy of dispersion and Coulomb interactions is slightly different. In such “sandwich” structure, the rotation of the rings of the molecules is impossible. However, in this case, the restriction of ThT fragment rotation does not lead to an increase in the fluorescence quantum yield on the order of 2–3, as for ThT in viscous solutions or for bound to amyloid fibrils.

This phenomenon can be explained using the framework of an exciton model^36–38^. The influence of the two molecules on each other splits an excited state ${S}_{1}^{\ast }$ with energy *Е*_1_ into two exciton states ${S}_{-1}^{\ast }$ and ${S}_{+1}^{\ast }$ with energies *E*_−1_ and *E*_+1_, respectively, with *E*_−1_ < *Е*_1_, and *E*_+1_ > *Е*_1_. According to exciton theory, in the case when two molecules form a sandwich-like structure (H-aggregates)^36^, the transition from the ground state to the state ${S}_{-1}^{\ast }$ is forbidden; therefore, photoexcitation leads to a transition to the state ${S}_{+1}^{\ast }$. In this case, the transition energy is greater than in the monomer case, which leads to the blue shift of the absorption spectrum. It is important to note that deactivation of the excited state ${S}_{+1}^{\ast }$ occurs through a non-radiative transition to the state ${S}_{-1}^{\ast }$, after which, a non-radiative transition to the ground state occurs. The blue shift of the absorption spectrum and the fluorescence quenching are specific features of H-aggregates. Nevertheless, the fluorescence quantum yield of excimers is an order of magnitude larger, and the fluorescence lifetime is 2 times larger (see, Fig. 3) than these values for the ThT monomer, which fluorescence quenching is determined by its molecular rotor nature.

**Figure 8:**
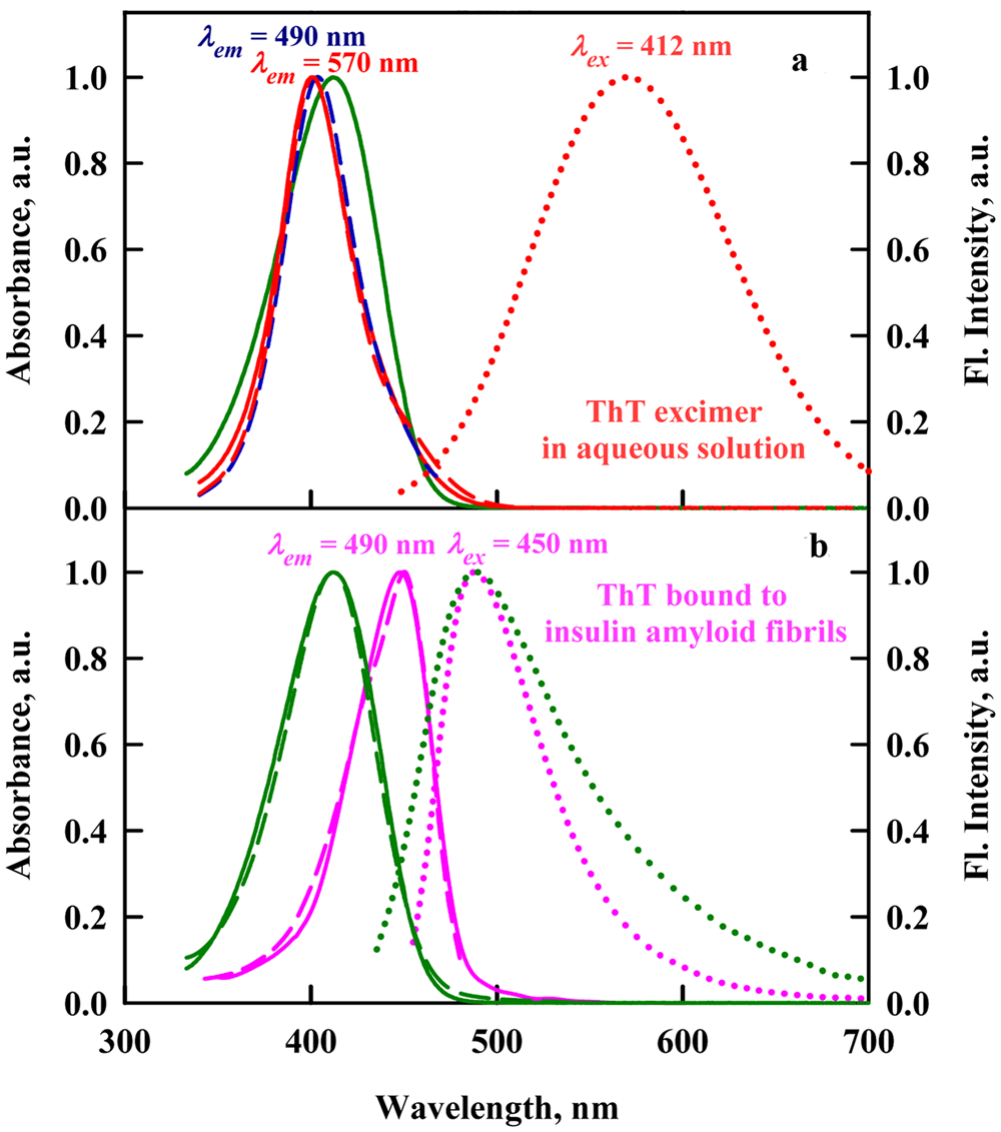
The absorption, fluorescence excitation and fluorescence spectra of ThT in aqueous solution and incorporated into amyloid fibrils. Panel a. Spectral characteristics of ThT in highly concentrated aqueous solution (ThT concentration was 28 mM). The absorption (red curve), fluorescence excitation (*λ*_*em*_ = 490 and 570 nm, blue and red curves, respectively) and fluorescence (*λ*_*ex*_ = 412 nm, red curve) spectra are presented. For comparison absorption spectrum of ThT in diluted aqueous solution (green curve) is given. Absorption spectrum, fluorescence excitation spectrum and fluorescence spectrum are given by solid, dashed and dotted lines, respectively. Panel b. Spectral characteristics of ThT bound to insulin amyloid fibrils. The absorption, fluorescence excitation (λ_em_ = 490 nm) and fluorescence (λ_ex_ = 450 nm) spectra are presented (magenta curves). Designations are the same as on the Panel A. Important, here is true absorption spectrum of ThT bound to amyloid fibrils obtained using solutions prepared by microdialyses after removal of free ThT absorption^49–52^, but not absorption spectrum of ThT in the presence of amyloid fibrils which is very close to absorption spectrum of free ThT molecules in aqueous solutions. For comparison the absorption, fluorescence excitation (λ_em_ = 490 nm) and fluorescence (λ_ex_ = 450 nm) spectra of free ThT in solution are presented (green curves).

**In conclusion**, for the first time, the fluorescence spectra of ThT in aqueous solutions in the broadest range of concentrations (from 3·10^−6^ to 3·10^−2^ M) at other conditions constant were recorded. Mostly important, for all concentrations scheme of registration was the same: fluorescence was registered at 90° to the excitation light propagation. Such measurements became possible due to the use Cary Eclipse spectrofluorimeter with horizontal slits and due to the developed in our laboratory method of correcting the fluorescence intensity^34^. Experimental data obtained for highly concentrated solutions of ThT filled the gap in our understanding of ThT spectral properties. It was shown that fluorescence band with maximum at 570 nm (Fig. 8, Panel a) revealed for highly concentrated ThT aqueous solutions can be attributed to ThT excimers. It was also shown that the ThT excimer fluorescence spectrum has nothing in common with the fluorescence of ThT incorporated into amyloid fibrils, which has maximа quite near that of ThT monomers in diluted solutions (Fig. 8, Panel b). Consequently, obtained data can be regarded as further confirmation of the idea that the increase in the ThT fluorescence intensity when the dye binds to amyloid fibrils can be explained only by the molecular rotor nature of ThT, and there is no basis to couple it with ThT excimers formation.

## Methods

### Absorption measurements

The absorption spectra of ThT (“UltraPure Grade” from AnaSpec, USA) were recorded using a U-3900H spectrophotometer (Hitachi, Japan). For recordings of the absorption spectra of the solutions over a wide range of concentrations, cells (Hellma Analytics, Germany) with the following different optical path lengths were used: 1, 0.5, 0.1, 0.01, and 0.001 cm. The concentration of ThT in solutions was determined using a molar extinction coefficient of *ε*_*412*_ = 31600 M^−1^cm^−1^ (according to the results of our measurements). Distilled water was used as the solvent.

### Fluorescence measurements

Fluorescent measurements were performed with a Cary Eclipse spectrofluorimeter (Agilent Technologies, Australia) in the cells 26.400FQ(Z20) 10 × 10 × 4 mm (Starna, USA).

The total fluorescence intensity $F({\lambda }_{ex})={\int }_{{\lambda }_{em}}F({\lambda }_{ex},{\lambda }_{em})d{\lambda }_{em}$, (where *F(λ*_*ex*_, *λ*_*em*_ *)* is the fluorescence intensity that is excited at the wavelength *λ*_*ex*_ and recorded at the wavelength *λ*_*em*_, and the fluorescence spectra were determined using 412 nm wavelength excitation light. The fluorescence excitation spectra were recorded at emission wavelengths of 490 and 570 nm. The spectral slits width was 10 nm. Changing the slits width did not influence the experimental results. The recorded values for total fluorescence intensity were corrected for the inner filter effect (see also Supplementary Method 1)^34^. PBS solutions of the fluorescent dyes N-acetyl-tryptophan amide (NATA) from Sigma-Aldrich (USA) and АТТО-425 from ATTO-TEC (Germany) were used without additional purification as a reference to normalize the recorded and corrected values of the ThT fluorescence intensity. These values are presented as the product of the absorbance and the fluorescence quantum yield of the dye. A value of 0.9 was used for the fluorescence quantum yield of АТТО-425 (ATTO-TEC Catalogue 2009/2010 p. 14) and 0.14 for NATA. All experiments were performed at room temperature (23 °C).

### Fluorescence intensity correction for inner filter effects

The fluorescence experiments were done with ThT aqueous solutions of a wide range of the dye concentration (3·10^−6^ to 3·10^−2^ M). Such experiments could not be done without using the spectrofluorimeter with horizontal slits^39, 40^ (see Supplementary Fig. S1) Cary Eclipse and fluorescence intensity correction on the primary inner filter effect^34^. For readers convenience a summary of this method is given in the Supplementary Method 2. At high concentrations of ThT in aqueous solutions fluorescence spectra of the monomer and excimer are distorted due to secondary inner filter effect: reabsorption of fluorescence in the short-wave part of the spectrum. The fluorescence spectrum measured for ThT solution with low concentration of the dye (with absorbance at 412 nm equal to 0.1, 0.2 or 0.3) was taken as true monomer fluorescence spectrum. At such concentrations the effect of secondary inner filter and excimer fluorescence are negligibly small. The fluorescence spectrum measured for ThT solution with high concentration of the dye (with absorbance at 412 nm equal to 600–880) was approximated by log-normal distribution (SigmaPlot 12.5) on the basis of the long-wavelength part of spectrum was taken as true excimer fluorescence spectrum. At such concentrations the contribution of monomer in the total fluorescence spectrum is negligibly small.

On the basis of the known shapes of monomer and excimer spectra, each recorded fluorescence spectrum was decomposed into these two components. The contribution of the monomer (*F*_*mon*_) and excimer (*F*_*exc*_) fluorescence in the recorded fluorescence spectra was determined in the spectral region where a fluorescence spectrum is not distorted to the secondary inner filter effect (λ_em_ > 550 nm). This was done for all solutions from 3·10^–6^ to 3·10^−2^ M (absorbance from 0.1 to 880) using multiple linear regression:

3 $$F({\lambda }_{em})={F}_{mon}({\lambda }_{em})+{F}_{exc}({\lambda }_{em})=a{{\rm{\Omega }}}_{mon}({\lambda }_{em})+b{{\rm{\Omega }}}_{exc}({\lambda }_{em}).$$

Here, Ω_*mon*_(*λ*_*em*_) and Ω_*exc*_(*λ*_*em*_) (${\rm{\Omega }}({\lambda }_{em})=F({\lambda }_{em})/\int F({\lambda }_{em})d{\lambda }_{em}$) are the shapes of the monomer and excimer fluorescence spectra, respectively. Using the determined coefficients *a* and *b*, we calculated ${F}_{mon}({\lambda }_{em})=a{{\rm{\Omega }}}_{mon}({\lambda }_{em})$, ${F}_{excim}({\lambda }_{em})=b{{\rm{\Omega }}}_{mon}({\lambda }_{em})$ and ${F}_{corr}({\lambda }_{em})={F}_{mon}({\lambda }_{em})+{F}_{exc}({\lambda }_{em})=a{{\rm{\Omega }}}_{mon}({\lambda }_{em})+b{{\rm{\Omega }}}_{exc}({\lambda }_{em})$ for each investigated solution.

### Time-resolved fluorescence measurements

Ultrafast fluorescence decays were measured using a femtosecond fluorescence up-conversion device (FOG100, CDP, Russia). The fluorescence up-conversion technique was employed to measure the time-resolved emission of ThT in aqueous solutions at concentrations of 3·10^−5^ and 1.5·10^−3^ M (absorbance ~1 and 50, respectively). For excitation, we used a cavity-dumped titanium:sapphire femtosecond laser TISSA-60, which provided short (<100 fs) pulses at a repetition rate of 85 MHz. The samples were excited with second harmonic light (420 nm), and the 490 and 570 nm fluorescence from the samples was up-converted by mixing it with the fundamental light pulse (gate pulse ~840 nm). The up-converted signal was measured using a photon counter after passing through a proper bandpass filter and a double monochromator. All of these measurements were performed in a rotating cell (1 mm path length) to ensure better heat dissipation and to avoid dye photodegradation. The measured emission decays were fit to a multiexponential function using the standard convolute-and-compare nonlinear least-squares procedure^41^. In this method, the convolution of the model exponential function with the instrument response function (IRF) was compared to the experimental data until a satisfactory fit was obtained. The IRF was measured using cross correlation of the excitation and fundamental gate pulse. A special program was used to analyze the decay curves^42^. The fitting routine was based on the nonlinear least-squares method. Minimization was performed according to Marquardt^43^.

### Quantum-chemical calculations

Quantum-chemical calculations of ThT dimer structures and energies were performed using the FireFly 8.0.1 software^44^. Dimer geometry optimization in the ground state was performed using density functional theory (DFT)^45^ corrected for the dispersion interaction (the Grimm correction)^46^ and using the Becke-Lee-Yang-Parr hybrid functional (B3 LYP)^47, 48^ and split valence basis set 3–21G.

## Electronic supplementary material

Thioflavin T fluoresces as excimer in highly concentrated aqueous solutions and as monomer being incorporated in amyloid fibrils
