# Supplementary material for: Thioflavin T fluoresces as excimer in highly concentrated aqueous solutions and as monomer being incorporated in amyloid fibrils
Source: Sci Rep. 2017 May 19;7:2146. doi: 10.1038/s41598-017-02237-7 (PMC5438369; doi:10.1038/s41598-017-02237-7)
Supplement: Supplementary file 1 — Thioflavin T fluoresces as excimer in highly concentrated aqueous solutions and as monomer being incorporated in amyloid fibrils [file 41598_2017_2237_MOESM1_ESM.pdf]

Thioflavin T fluoresces as excimer in highly concentrated aqueous solutions and as monomer being incorporated in amyloid fibrils

Anna I. Sulatskaya,[1] *Andrey V. Lavysh,*[2] *Alexander A. Maskevich,*[2] *Irina M. Kuznetsova,*[1] *Konstantin K. Turoverov*[1,3,*]

[1]*Laboratory for structural dynamics, stability and protein folding, Institute of Cytology Russian Academy of Science, St. Petersburg 194064, Russia;*
[2]*Department of Physics, Yanka Kupala Grodno State University, Grodno, 230023, Belarus;*
[3]*Department of Biophysics, St. Petersburg State Polytechnic University, St. Petersburg, 195251, Russia*
*E-mail: kkt@incras.ru

Supplementary Information

Table of contents

Supplementary Method 1. Fluorescence intensity correction for the primary inner filter effect.. 2

Supplementary Method 2. Particularities of Cary Eclipse spectrofluorimeter.......... 3

Supplementary Figure S1. Schematic representation of the light flux in spectrofluorimeters with vertical (standard) and horizontal slit geometries................. 4

Supplementary Figure S2. Fluorescence spectra of ThT in aqueous solutions with different absorbances (different concentrations) after correction for the primary and both primary and secondary inner filter effects.. 5

Supplementary Figure S3. The fluorescence spectra of ThT aqueous solutions of different concentrations with corrections for the primary and secondary inner filter effects and normalization to the total fluorescence intensity...................................... 6

Supplementary References.. 6

Supplementary Method 1

Fluorescence intensity correction for the primary inner filter effect

The nonlinearity of the concentration dependence of the fluorescence intensity is caused by the so-called primary inner filter effect. The reasons for this effect include both the attenuation of the excitation light flux on its path through an absorbing solution (Beer–Lambert law) and the difference between the area that is illuminated by the excitation light and the working area from which the fluorescence light is gathered.

It is generally accepted that for low concentration solutions (low absorbance), the fluorescence intensity is proportional to the concentration of the fluorescence substance, and primary inner filter effects are negligible; however, this assumption is not valid.[1] In reality, the total fluorescence intensity is only proportional to the absorbance (A) at one point, when $A = 0$. Even at $A = 0.1$, the deviation from linearity is 12%, and at $A = 0.3$, the deviation is 38%.

In an ideal case when the area illuminated by the excitation light coincides with the working area from which the fluorescence light, the recorded total fluorescence intensity

$$F(\lambda_{ex}) = \int_{\lambda_{em}} F(\lambda_{ex}, \lambda_{em}) d\lambda_{em} \qquad (1)$$

where $F(\lambda_{ex},\lambda_{em})$ is the fluorescence intensity that is excited at the wavelength λ_{ex} and recorded at the wavelength λ_{em}, is proportional to the fraction of the excitation light that is absorbed by the solution ($1-10^{-A_\Sigma}$). If only one substance is responsible for the absorption and fluorescence of a solution, then:

$$F(\lambda_{ex}) = k' I_0(\lambda_{ex}) \Delta\lambda_{ex} \left(1-10^{-A_\Sigma}\right) q = k \frac{\left(1-10^{-A_\Sigma}\right)}{A_\Sigma} A_{FL} q \qquad (2)$$

Here, $I_0(\lambda_{ex})\Delta\lambda_{ex}$ is the intensity of the excitation light at λ_{ex}, k' is a proportionality factor, $\Delta\lambda_{ex}$ is the spectral width of the slits of the monochromator in the excitation pathway, and $k = k' I_0(\lambda_{ex})\Delta\lambda_{ex}$ is a normalization factor determined using a standard (a fluorescent substance with known fluorescence quantum yield) at the same experimental conditions (i.e., slits widths, photomultiplier voltage, and other factors) that were used in the experiment with the sample. The coefficient k is chosen such that the total fluorescence intensity of the standard and the sample give physical meaning to the product of absorbance and the fluorescence quantum yield:

$$F_0(\lambda_{ex}) = F(\lambda_{ex})/W = A_{FL} q \qquad (3)$$

Here, W is a correction factor that is determined by the total absorbance of the solution at λ_{ex}:

$$W = \frac{1 - 10^{-A_{FL}}}{A_{FL}}. \qquad (4)$$

If there are several components in the solution that absorb $A_{FL,i}$ and fluoresce with a fluorescence quantum yield q_i, but others only absorb the excitation light with total absorbance A_{ABS}, then:

$$F_0(\lambda_{ex}) = F(\lambda_{ex})/W = \Sigma A_{FL,i} q_i \qquad (5)$$

where $W = \frac{1 - 10^{-A_\Sigma}}{A_\Sigma}$ and $A_\Sigma = \sum A_{FL,i} + A_{ABS}$.

The fluorescence excitation spectra recorded at 490 and 570 nm and corrected on the primary inner filter effect using the calculated coefficient W are shown in Fig. 2, Panels b and c. Obviously, both fluorescence excitation spectra recorded at 490 nm and at 570 nm are close to the corresponding absorption spectrum. Therefore, there are reasons to suggest that the calculated correction factor W could be used over a wider range of absorbance, than it was shown for NATA solutions of different concentration.[1]

Supplementary Method 2

Particularities of Cary Eclipse spectrofluorimeter

In most spectrofluorimeters, the detected fluorescence intensity is not proportional to the fraction of light that is absorbed by the solution. Thus, the correction factor cannot be calculated according to Eq. 4; instead, it must be determined experimentally.[1] Moreover, because the fluorescence intensity measured by these spectrofluorimeters decreases as the absorbance of the investigated solutions increases, the fluorescence of solutions with high absorbance (A > 5.0 for most spectrofluorimeters with a cell with an optical path length of 1 cm) cannot be recorded at all.

We showed experimentally[1] that the Cary Eclipse spectrofluorimeter enables recordings of fluorescent solutions with very high absorbance; in contrast to all known spectrofluorimeters, this spectrofluorimeter has horizontal slits (Fig. S1).[2] Using this spectrofluorimeter and rectangular cells with 10 mm optical path lengths in the present work, we recorded the fluorescence spectrum of a solution with absorbance A_{412}=880 at an excitation wavelength of 412 nm. The unique feature of this spectrofluorimeter is that the area illuminated by the excitation light coincides with the working area from which the fluorescence light is gathered (Fig. S1). Thus, in this spectrofluorimeter, the detected fluorescence intensity is proportional to the fraction of the light absorbed by the fluorescent solution. Consequently, the correction factor W

can be calculated using only the solution absorbance according to Eq. 4 for a wide range of absorbance values.

The use of the Cary Eclipse spectrofluorimeter allowed us to reliably measure the fluorescence spectra of solutions with very high concentrations (up to $3 \cdot 10^{-2}$ M, corresponding to absorbance values of approximately 10^{3}). The fluorescence spectra of ThT in aqueous solution at concentrations from $3 \cdot 10^{-6}$ to $3 \cdot 10^{-2}$ M, corresponding to absorbance values from 0.1 to 880 are shown in Figure 1

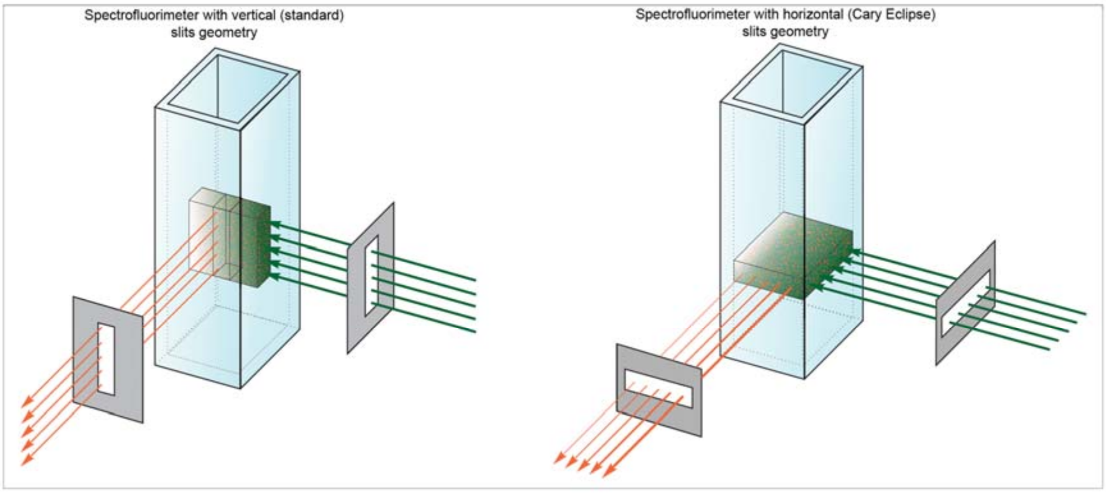

Figure S1. Schematic representation of the light flux in spectrofluorimeters with vertical (standard) and horizontal slit geometries.[3] The Figure is taken according to Plos ONE licenses (https://creativecommons.org/licenses/by/4.0/) from the paper by Fonin et al.[1]

.

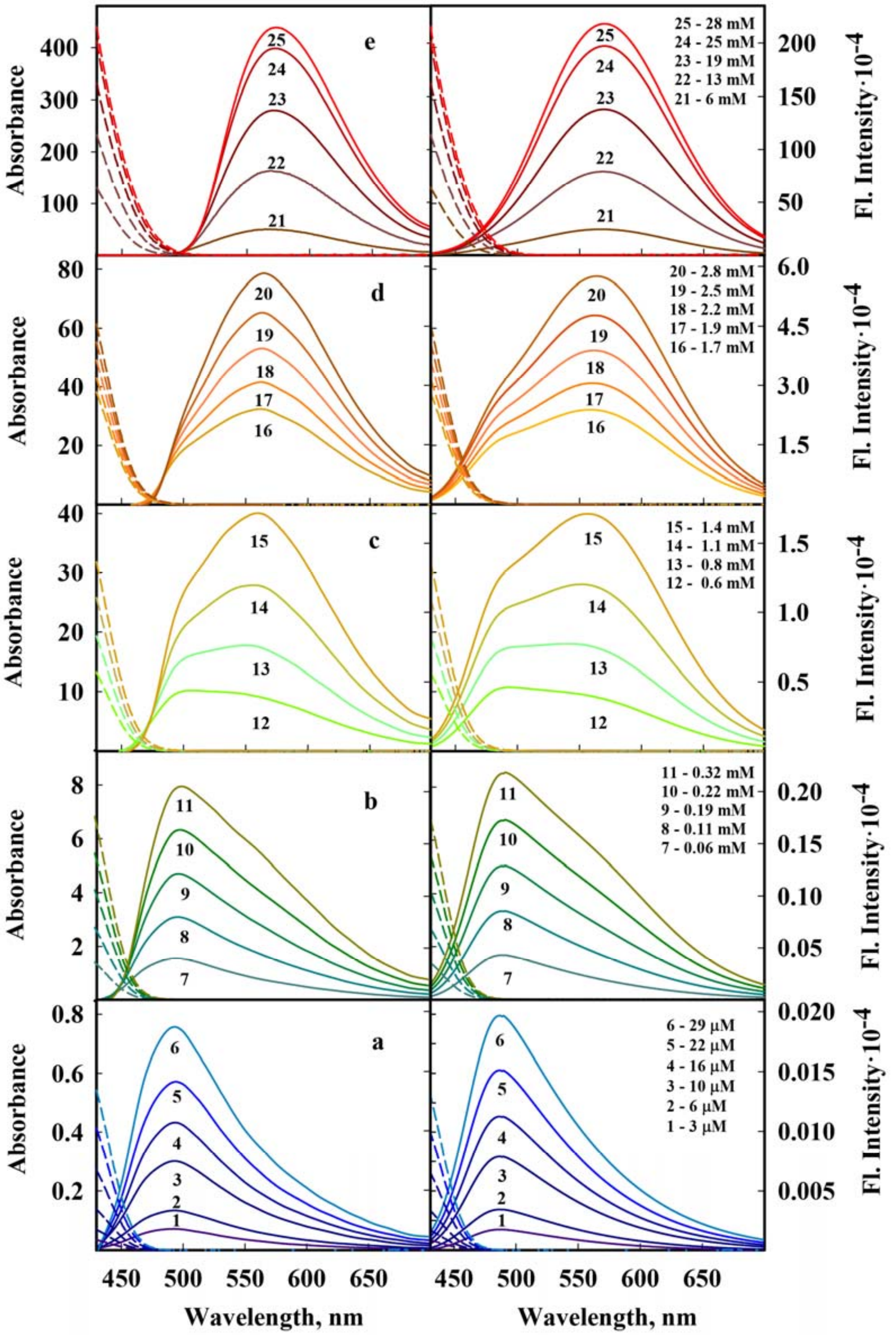

Figure S2. Fluorescence spectra of ThT in aqueous solutions with different absorbances (different concentrations) after correction for the primary and both primary and secondary inner filter effects. Left Panels. Fluorescence spectra corrected for primary inner filter effect. Right Panels. Fluorescence spectra corrected for both primary and secondary inner filter effects. Panels A-E show the fluorescence spectra of ThT with absorbance ranging from 0.1 to 1, from 2 to 10, from 20 to 50, from 60 to 90 and from 100 to 880, respectively, λ_{ex} =412 nm. The dotted curves are the red edges of the absorption spectra. The spectral slits width was 10 nm.

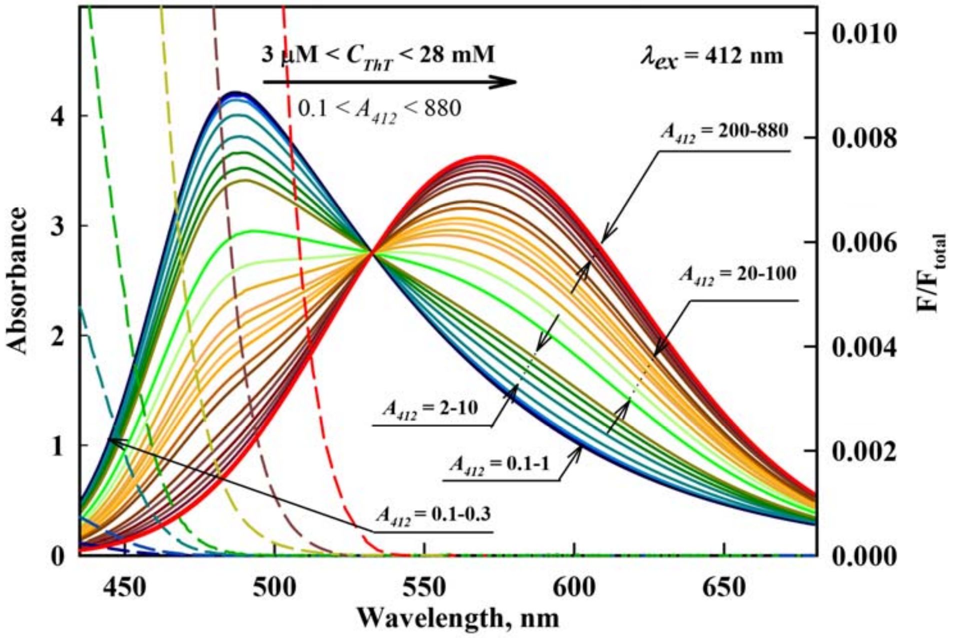

Figure S3. The fluorescence spectra of ThT aqueous solutions of different concentrations with corrections for the primary and secondary inner filter effects and normalization to the total fluorescence intensity. The ThT concentration was changed from 3 μM to 28 mM, which corresponds to absorbance changes from 0.1 to 880. λ_{ex} = 412 nm. The spectral slits width was 10 nm. The dashed curves represent the long wavelength edge of absorption spectra of solutions with concentrations 0.006, 0.016, 0.11, 0.32, 1.1, 6, 28 mM. Curves with A_{412}= 0.1 – 0.3 correspond to ThT concentrations 3, 6, 10 μM, curves with A_{412}= 0.1 – 1.0 correspond to ThT concentrations 3, 6, 10, 16, 22, 29 μM, curves with A_{412}= 2 – 10 correspond to ThT concentrations 0.06, 0.11, 0.19, 0.22, 0.32 mM, curves with A_{412}= 20 – 100 correspond to ThT concentrations 0.6, 0.8, 1.1, 1.4, 1.7, 1.9, 2.2, 2.5, 2.8 mM, curves with A_{412}= 220 – 880 correspond to ThT concentrations 6, 13, 19, 25, 1.7, 28 mM.

References

1 Fonin, A. V., Sulatskaya, A. I., Kuznetsova, I. M. & Turoverov, K. K. Fluorescence of dyes in solutions with high absorbance. Inner filter effect correction. *PLoS One* **9**, e103878, doi:10.1371/journal.pone.0103878 (2014).

2 Jameson, D. M., Croney, J. C. & Moens, P. D. Fluorescence: basic concepts, practical aspects, and some anecdotes. *Methods Enzymol* **360**, 1-43 (2003).

3 Steensrud, J. Selecting the correct spectrophotometer for quality results. *Agilent Technologies*, 25 (2012).
